# Discovery and validation of Ferroptosis-related molecular patterns and immune characteristics in Alzheimer’s disease

**DOI:** 10.3389/fnagi.2022.1056312

**Published:** 2022-11-23

**Authors:** Yi-Jie He, Lin Cong, Song-Lan Liang, Xu Ma, Jia-Nan Tian, Hui Li, Yun Wu

**Affiliations:** Department of Neurology, The Second Affiliated Hospital of Harbin Medical University, Harbin, China

**Keywords:** Alzheimer’s disease, immune characteristics, Ferroptosis, biomarkers, cluster, machine learning, diagnostic model, Nomogram

## Abstract

**Background:**

To date, the pathogenesis of Alzheimer’s disease is still not fully elucidated. Much evidence suggests that Ferroptosis plays a crucial role in the pathogenesis of AD, but little is known about its molecular immunological mechanisms. Therefore, this study aims to comprehensively analyse and explore the molecular mechanisms and immunological features of Ferroptosis-related genes in the pathogenesis of AD.

**Materials and methods:**

We obtained the brain tissue dataset for AD from the GEO database and downloaded the Ferroptosis-related gene set from FerrDb for analysis. The most relevant Hub genes for AD were obtained using two machine learning algorithms (Least absolute shrinkage and selection operator (LASSO) and multiple support vector machine recursive feature elimination (mSVM-RFE)). The study of the Hub gene was divided into two parts. In the first part, AD patients were genotyped by unsupervised cluster analysis, and the different clusters’ immune characteristics were analysed. A PCA approach was used to quantify the FRGscore. In the second part: we elucidate the biological functions involved in the Hub genes and their role in the immune microenvironment by integrating algorithms (GSEA, GSVA and CIBERSORT). Analysis of Hub gene-based drug regulatory networks and mRNA-miRNA-lncRNA regulatory networks using Cytoscape. Hub genes were further analysed using logistic regression models.

**Results:**

Based on two machine learning algorithms, we obtained a total of 10 Hub genes. Unsupervised clustering successfully identified two different clusters, and immune infiltration analysis showed a significantly higher degree of immune infiltration in type A than in type B, indicating that type A may be at the peak of AD neuroinflammation. Secondly, a Hub gene-based Gene-Drug regulatory network and a ceRNA regulatory network were successfully constructed. Finally, a logistic regression algorithm-based AD diagnosis model and Nomogram diagram were developed.

**Conclusion:**

Our study provides new insights into the role of Ferroptosis-related molecular patterns and immune mechanisms in AD, as well as providing a theoretical basis for the addition of diagnostic markers for AD.

## Introduction

Dementia is an acquired, progressive cognitive impairment that primarily affects the ability to perform everyday tasks and is a significant cause of dependency, disability and death in older people (Lane et al., 2018). Epidemiological surveys show that the global prevalence of dementia was around 50 million people in 2018, which is expected to triple by 2050 (Scheltens et al., 2021). Alzheimer’s disease (AD) is a multifactorial neurodegenerative disorder. It is the most common form of dementia, manifesting primarily as cognitive, emotional, language and memory impairments in the elderly, first identified and reported by Alzheimer (1907), Alzheimer et al. (1995), and Sabayan and Sorond (2017). Perennial deposits of amyloid β (Aβ) and neuronal fibrillary tangles (NFTs) are thought to be prominent features in the pathogenesis of AD (Schneider and Sari, 2014). However, the cellular and molecular mechanisms that contribute to the pathogenesis of AD have not been fully elucidated.

Ferroptosis is an iron-dependent form of cell death that differs from other cell death in that its mechanisms include apoptosis and necrosis. Specifically, the process involves the three main metabolisms of thiols, lipids and iron, leading to the production of iron-dependent lipid peroxidation and, ultimately, cell death (Yan et al., 2021). In contrast to other tissues and organs, human brain tissue is rich in iron, which plays a vital role in various physiological processes, including DNA synthesis, neurotransmitter synthesis, and metabolism (Ward et al., 2014). Moreover, brain tissue is rich in PUFA (polyunsaturated fatty acids) and iron compared to other tissues and organs in the body. As a result, it consumes more oxygen, is more susceptible to lipid peroxidation and has a high susceptibility to iron death (Ma et al., 2022). Evidence from the previous studies (Smith et al., 2010; Liu et al., 2011) suggests that iron may also bind to and cause aggregation of Aβ and Tau proteins, which also predicts the possibility of the Ferroptosis mechanism as potential pathogenesis of AD. As research continues, Ferroptosis is increasingly being recognised as a distinct mechanism of cell death in the pathogenesis of AD (Chen et al., 2021; Jakaria et al., 2021; Ma et al., 2022). Furthermore, Ferroptosis is regulated by a set of genes, and detecting these genes as evidence of iron death is essential for further studies exploring the pathogenesis of AD.

On the other hand, there has been experimental evidence that neuroinflammation and immune responses play a critical role in the pathogenesis of AD (Morales et al., 2014). Among other things, it has been shown that Aβ deposition can drive the migration of inflammatory factors and microglia to affected sites, exacerbating the inflammatory response and promoting plaque formation through the induction of cell death (Simard et al., 2006; Baik et al., 2016). Further experimental evidence suggests that the continued activation of the immune response indicates the accumulation of more microglia, making the Aβ deposition-immune response a positive feedback loop that further exacerbates the pathological changes of neuroinflammation and AD (Hickman et al., 2008). And recent studies (Long et al., 2022) point to a complex regulatory relationship between microglia, iron, and immune inflammation, which play a role in AD development. Fernández-Mendívil et al. (2020) found that under conditions of neuroinflammation, microglia can lead to the toxic accumulation of iron by upregulating the gene HO-1, which increases the production of reactive oxygen species (ROS), ultimately leading to the development of memory impairment in AD mice. Another study in GPX4-deficient mice (Hambright et al., 2017) showed that Ferroptosis-related markers could induce learning and memory deficits in mice by regulating increased lipid peroxidation and neuroinflammatory responses. All of the above findings suggest a potential role for Ferroptosis mechanisms and immune responses in the pathogenesis of AD. However, there are relatively few bioinformatics studies on the mechanisms of Ferroptosis in the field of AD, and a comprehensive analysis is still lacking.

**FIGURE 1:**
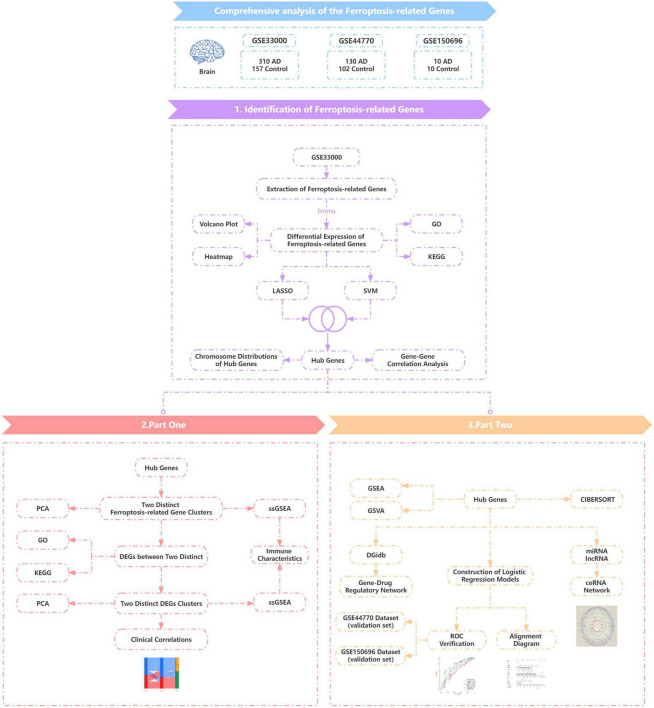
Technology route. AD, Alzheimer’s disease; Control, healthy control.

This study aimed to comprehensively analyse and explore the molecular mechanisms of Ferroptosis-related genes in the pathogenesis of AD as well as the immunological features (Figure 1).

## Materials and methods

### Identification of Ferroptosis-related genes

#### Download and processing of expression spectrum data

In this study, we downloaded the gene expression profile datasets GSE33000, GSE44770 and GSE150696 of AD from the GEO database (Table 1). The dataset GSE33000 was published publicly in 2014 by Narayanan et al. (2014). The study demonstrated that a shared dysregulated network in the human prefrontal cortex underlies two neurodegenerative diseases. The data sample used in the study was obtained from the HBTRC (Harvard Brain Tissue Resource Center) and included 157 healthy controls and 310 AD patients. The postmortem interval (PMI) of samples in the entire cohort was 17.8 ± 8.3 h, the sample PH was 6.4 ± 0.3, and the RNA integrity (RIN) was 6.8 ± 0.8. The above samples were analysed by Agilent 44K arrays containing 40,638 DNA probes targeting 39,909 mRNA transcripts for 19,198 known genes and 20,711 predicted genes. The dataset GSE44770 was publicly available in 2013. Zhang et al. (2013) developed and applied an integrated network-based approach to identify gene targets associated with neurodegenerative diseases by analysing their data. The tissue sources for this dataset included the dorsolateral prefrontal cortex (PFC), visual cortex (VC), and cerebellum (CB). Samples of PFC origin were selected for further analysis, involving 130 AD patients and 102 healthy controls. Tissue samples were analysed by Rosetta/Merck Human 44k 1.1 microarrays for 39,579 transcripts, including 25,242 known genes and 14,337 predicted genes. The cohort’s PMI, PH and RIN samples were 17.8 ± 8.3h, 6.4 ± 0.3 and 6.8 ± 0.8, respectively. The dataset GSE150696 was made publicly available in a 2013 study by Low et al. (2021), which suggested that FynT may be involved in the pathological alterations of AD through its effects on tauopathy and neuroinflammation. The prefrontal cortex sample used in this dataset was provided by Brains for Dementia Research (BDR). Samples were analysed by Affymetrix Human Transcriptome Array 2.0. Age, gender and PMI were matched as much as possible during the analysis. In addition, we integrated the clinical information of the patients selected in the dataset, including age, gender, and disease status (Supplementary Data Sheet 10).

**TABLE 1:** Dataset information from the GEO database.

Location	Accession	Platform	Type	Number
Brain	GSE33000	GPL4372	Microarray	157 control vs. 310 AD
Brain	GSE44770	GPL4372	Microarray	102 control vs. 130 AD
Brain	GSE150696	GPL17585	Microarray	10 control vs. 10 AD

The next step is to pre-process the downloaded dataset. Specifically, two components are included: ID conversion of the probe data and normalisation of the expression spectrum matrix. The ID conversion of probe data is performed by a Perl language script,^1^ which first obtains the gene’s name corresponding to each probe from the downloaded platform annotation file. It converts the probe name to the corresponding gene ID to complete the annotation. We observed multiple probes corresponding to the same gene ID during annotation. At this point, we averaged the data of all probes corresponding to the gene, and the processed data were used as the expression profile data of the gene. The normalizeBetweenArrays function does the normalisation of the expression spectrum data.

#### Extraction of Ferroptosis-related genes

The set of Ferroptosis-related Genes used in this study was extracted from the FerrDb database (Zhou and Bao, 2020), the first database to provide Ferroptosis-related markers, modulators, and all Genes in this data were experimentally validated. Therefore, we downloaded the Ferroptosis-related gene set from FerrDb and extracted the expression of these genes in the dataset GSE33000 to construct the Ferroptosis-related Genes expression profile matrix for subsequent analysis.

#### Differential expression analysis

We performed differential expression analysis of the Ferroptosis-related Genes expression profile matrix for identifying Ferroptosis-related differentially expressed genes (FRDEGs) between the AD and Con groups. The significance criterion for FRDEGs was set at *P* < 0.05. Based on this, the expression of FRDEGs was visualised and analysed, the heatmap of FRDEGs was constructed using the pheatmap package, and the ggplot2 package implemented the volcano map. Finally, gene ontology (GO) and Kyoto Encyclopedia of Genes and Genomes (KEGG) analyses were performed on FRDEGs, where GO enrichment analysis included three parts (BP, CC, MF) to observe the function or pathway of FRDEGs enrichment.

#### Construction of LASSO model and mSVM-RFE feature selection process

Two machine learning algorithms, least absolute shrinkage and selection operator (LASSO) logistic regression and multiple support vector machine recursive feature elimination (mSVM-RFE) algorithm, were used to identify the genes most associated with AD. The LASSO logistic regression analysis was performed using the “glmnet” package with the following parameters: response type set to binomial, alpha set to 1, and 10-fold cross-validation to adjust the optimal value of the parameter λ. In addition, SVM-RFE serves as a practical feature selection technique to find the best variables by removing the SVM-generated feature vectors. Still, the drawback is that the pure SVM-RFE algorithm is an algorithm based on the binary case, which can reduce the performance of gene selection. The mSVM-RFE algorithm used in this study is similar to the SVM-RFE step, which is still constructed by the “e1071” package, except that the mSVM-RFE algorithm calculates the ranking score of a gene at each stage, which is calculated statistically from the weight vector coefficient. The specific parameters are set as follows: halfve. above = 50 and *k* = 10, using 10-fold cross-validation to make the algorithm more accurate. The common genes identified by the two machine learning methods were the Hub genes for the subsequent study. Correlation analysis of the Hub genes identified by the two machine learning algorithms was performed and visualised using the “corrplot” package. The “RCircos” package was also used to plot circles to show the distribution of Hubs on chromosomes. Finally, the expression levels of Hub genes in various human tissues were analysed using the GETx database.

### Part one

#### Gene cluster based on Ferroptosis-related gene expression

The analysis of the AD dataset was implemented in R using the “ConsensusClusterPlus” software package (Wilkerson and Hayes, 2010), which identifies possible groups of shared biometric traits based on Hub genes, defined as “FRG Cluster.” A resampling-based approach is used to assess the plausibility and stability of consensus clustering. Clusters sort the Consensus Matrix graph to find the “cleanest” clusters distribution, showing high consensus (dark blue) for items with high cohesion and low consensus for items with low cohesion. The empirical cumulative distribution function (CDF) represents what value of K is taken when the cluster analysis results are most reliable, and the CDF value is approximately the maximum value. The Delta plot represents the area under the CDF curve, which corresponds to the relative change in k and k-1. Specifically, the optimal number of clusters is chosen based on the heat map of the Consensus Matrix, the empirical cumulative distribution function (CDF), and the delta map.

Principal component analysis (PCA) was performed on the Hub genes identified by the machine learning algorithm to visualise the typing results further.

#### Correlations between two distinct clusters of Ferroptosis-related genes and immune characteristics

To determine the correlation between the two molecular typologies and the immune microenvironment, we applied the ssGSEA method (Xiao et al., 2020) to analyse the proportion of 28 different immune cell distributions and infiltration scores for each sample in both typologies. Using this approach, the extent of immune cell infiltration in each sample was determined, and the correlation between Ferroptosis-related gene expression and the amount of immune cell infiltration was determined. Heatmaps and box line plots were created to visualise the analysis results using the “Pheatmap” and “Voplot” packages.

#### Identification of differential expression genes between two distinct clusters

We performed intergroup differential expression analysis of Two Distinct Clusters of Ferroptosis-related Genes obtained from the above steps to get the differentially expressed genes (CDEGs) between the two Clusters. Based on the CDEGs, we applied the “ConsensusClusterPlus” package to create a new genotype, defined as a “gene Cluster.”

Secondly, the obtained CDEGs were enriched and analysed to observe their possible involvement in biological functions or pathways. The enrichment analysis consists of the Gene Ontology (GO) and the Kyoto Encyclopedia of Genes and Genomes (KEGG). Finally, the same ssGSEA algorithm was used for each sample to obtain an infiltration score of 28 immune cells to analyse their degree of infiltration. Further correlation analysis was used to determine the association of CDEGs gene expression with immune cell infiltration.

#### Correlation of FRGscore with clinical and immune characteristics

Using the PCA method, FRG scores were generated based on the expression profiles of 10 Ferroptosis-related Genes (Hub genes) in AD samples. Specifically, PC1 and PC2 were extracted to form signature scores, and FRGscore was constructed by applying a method similar to the Gene Expression Index (GGI) (Sotiriou et al., 2006; Li S. et al., 2022):


(1) F⁢R⁢G⁢s⁢c⁢o⁢r⁢e=∑(P⁢C⁢1⁢i+P⁢C⁢2⁢i)

Where *i* denotes the expression of the Hub gene. The FRGscore was compared between two different molecular typing (FRG cluster and gene Cluster). We further analysed the relationship between the FRG cluster, gene Cluster, Age and FRGscore shown as Sanky plots. Correlations between FRGscore and immune cells and responses were determined using Pearson’s correlation analysis to visualise the results using a correlation heatmap.

### Part two

#### Correlation analysis between Hub genes and immune characteristics

CIBERSORT is currently the most cited tool for immune cell infiltration analysis. It is based on linear support vector regression to calculate the gene expression signature set of 22 immune cell subtypes: LM22. In this study, we calculated the infiltration scores of 22 immune cells for each sample in the dataset GSE33000 using the CIBERSORT algorithm. We analysed the correlation between Hub genes and immune cells to visualise the analysis results graphically.

#### Enrichment analysis of Hub genes

Gene set variance analysis (GSVA) is a non-parametric, unsupervised analysis based on a list of applicable terms or gene sets, where pathway enrichment is evaluated for each sample. We applied the “GSVA” package to complete the above process analysis in this study. Gene set enrichment analysis (GSEA) is a computational method of the functional class scoring approach to identify whether pre-selected gene sets are differentially expressed between groups. The available pathways of Hub genes were predicted and analysed using two methods of enrichment analysis.

#### Construction of gene-drug regulatory networks

The Drug-Gene Interaction Database^2^ is an online database of drug-gene interaction data mined from DrugBank, PharmGKB, Chembl, Drug Target Commons, TTD, and other databases. The list of Hub genes was imported into the website to get the interaction score, nature of interaction and interaction information of different drugs for the gene. Finally, the above information is used to construct a gene-drug regulatory network to find potential drug targets.

#### Construction of the competitive endogenous RNA regulatory network

RNAs can regulate each other by competing for binding a common miRNA, a mode of regulation known as competitive endogenous RNA (ceRNA). The ceRNAs identified include protein-coding mRNAs and non-coding RNAs, the latter including lncRNAs and cicrRNAs. In the present study, we constructed a ceRNA regulatory network between mRNA-miRNA-lncRNA. Specifically, three databases, miRanda, miRDB and TargetScan, were used to simultaneously predict the target miRNAs of Hub genes and take the intersection. Obtain mRNA-miRNA files. The second step uses the result file from the previous step to predict the lncRNAs it targets through the spongeScan database. Finally, the ceRNA network is constructed, and the feature files are jointly built based on the lncRNA-miRNA-mRNA files, the classification of genes and the regulation relationship obtained above. Visualise ceRNA regulatory networks using Cytoscape.

#### Development and validation of a diagnostic model for Ferroptosis-related genes

Based on 10 Hub genes, a logistic regression algorithm was used to build a diagnostic model for AD classification. The area under the ROC curve (AUC) was used to assess the accuracy of the Hub gene and logistic regression models. Therefore, we calculated the AUC values of the 10 Hub genes and logistic regression models separately to assess the accuracy of the diagnostic model. In addition, we calculated the AUC values of the Hub gene and logistic regression models in dataset GSE44770 (containing 130 AD patients and 102 normal controls) and dataset GSE150696 (having 10 AD patients and 10 normal controls), respectively, as a way to validate the classification performance of the diagnostic models. Finally, the visual analysis of the above process is completed using ggplot2.

#### Modeling and validation of a diagnostic Nomogram

Using 10 Hub genes, a column line graph was constructed based on the “rms” R package. The accuracy of the column line graphs was estimated using calibration curves, and the clinical significance of the column line graphs was evaluated using decision curve analysis.

## Results

### Identification of Ferroptosis-related genes

#### Identification of differential expression of Ferroptosis-related genes

**FIGURE 2:**
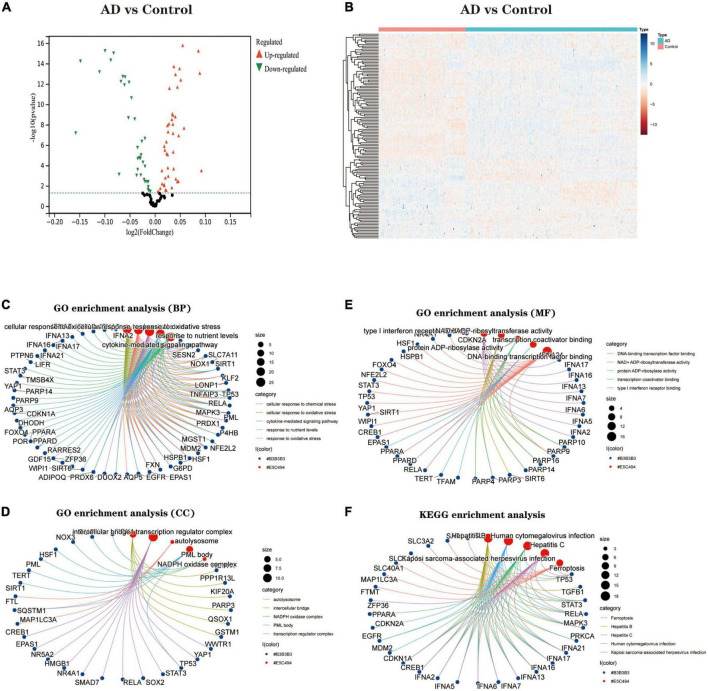
**(A)** The differential expression analysis results are shown in the volcano plot. The x-axis represents log2 (fold change), and the y-axis represents -log10 (adjust *p*-value). Green triangles represent downregulated genes, red triangles represent upregulated genes, and black dots represent genes with no evident differential expression. **(B)** Heatmap of FRDEGs. Each column in the graph represents a sample, each row represents a gene, and the expression status of the gene is indicated from high to low in orange to blue, respectively, and at the top of the heat map, blue/red represents the AD group/control group, respectively. **(C)** Shows the top 5 significantly enriched BP (biological process). **(D)** Shows the top 5 enriched CC (cellular component) considerably. **(E)** The top 5 enriched MF much (molecular function) are shown. **(F)** The top 5 significantly enhanced KEGG pathways. In the bubble plots for GO and KEGG enrichment analysis, blue dots represent genes, red dots represent pathways, and bubble size represents the number of genes enriched in that pathway. AD, Alzheimer’s disease; Control, healthy control; FRDEGs, Ferroptosis-related differentially expressed genes; GO, Gene Ontology; KEGG, Kyoto Encyclopedia of Genes and Genomes.

We downloaded the Ferroptosis-related gene set from FerrDb, with 728 genes. The expression of these genes was extracted to build a Ferroptosis-related gene expression matrix. A total of 138 FRDEGs (Supplementary Data Sheet 1) were identified by differential expression profiling of 310 brain tissue samples from AD patients and 157 normal human brain tissue samples, which included 67 up-regulated and 71 down-regulated genes (Figures 2A,B). We further explored the possible signalling pathways involved in FRDEGs by GO with KEGG enrichment analysis (Supplementary Data Sheet 2). The GO results indicated that the above genes are involved in the cellular response to oxidative stress, cellular response to chemical stress, and response to oxidative stress BP pathways (Figure 2C). For the CC pathway (Figure 2D), FRDEGs function primarily in the intercellular bridge and transcription regulator complex. The functional enrichment of MF (Figure 2E) showed that FRDEGs are mainly involved in molecular functions such as DNA-binding transcription factor binding and DNA-binding transcription factor binding. The KEGG (Figure 2F) analysis favoured Ferroptosis and Human cytomegalovirus infection pathways.

#### Identification of Hub genes

**FIGURE 3:**
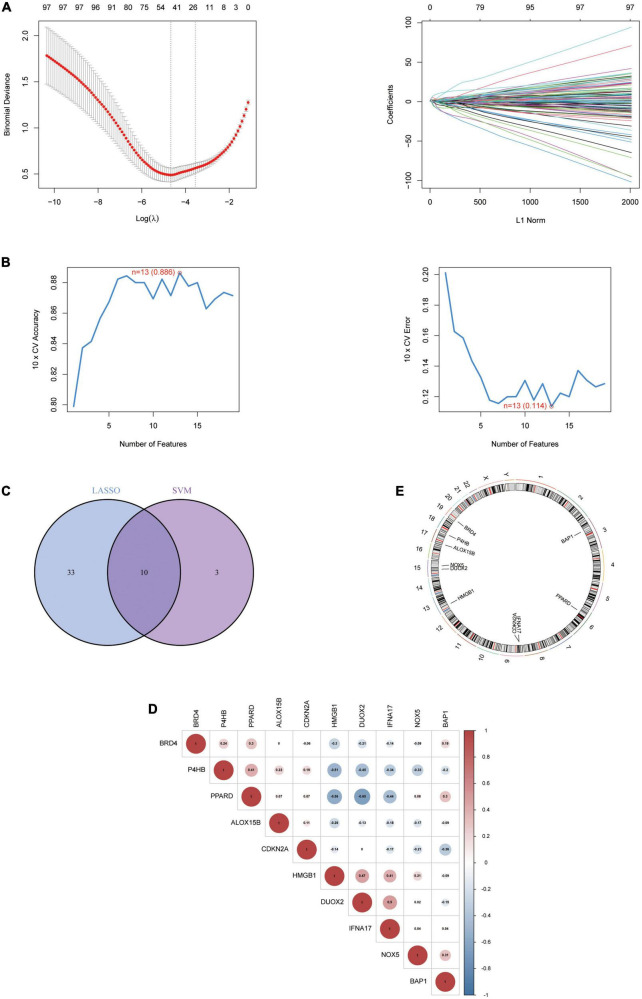
The identification of 10 Hub genes. **(A)** The LASSO logistic regression algorithm identified 43 AD-related features with a 10-fold cross-validation set for selecting the penalty parameter to determine the optimal lambda value. **(B)** A total of 13 feature genes were filtered out using the mSVM-RFE algorithm. **(C)** Venn diagram of genes extracted from LASSO and mSVM-RFE methods. **(D)** Heatmap of correlations for Hub genes. Positive correlations are marked in red and negative correlations are marked in blue. The numbers in the middle represent correlation coefficients. **(E)** Map of the location of the Hub gene on the chromosome. LASSO, least absolute shrinkage and selection operator; SVM, support vector machine; RFE, recursive feature elimination.

To explore biomarkers of AD, we screened genes using two different machine learning algorithms. The results of the LASSO regression showed (Figure 3A) that 43 genes were considered the most associated with AD (Best λ for LASSO: 0.009376). On the other hand, the mSVM-RFE algorithm (Figure 3B) yielded 13 genes. Both machine algorithms were subjected to 10-fold cross-validation to ensure the accuracy of the results. The common gene identified by the two machine learning methods (Figure 3C) was the hub gene for the subsequent study. We further analysed the correlation between Hub genes (Figure 3D) and showed the distribution of Hub genes on the chromosomes (Figure 3E). Finally, the expression levels of Hub genes in various human tissues were analysed using the GETx database (Supplementary Figures 1 and 2).

### Part one

#### Results based on two distinct clusters of Ferroptosis-related gene expression

**FIGURE 4:**
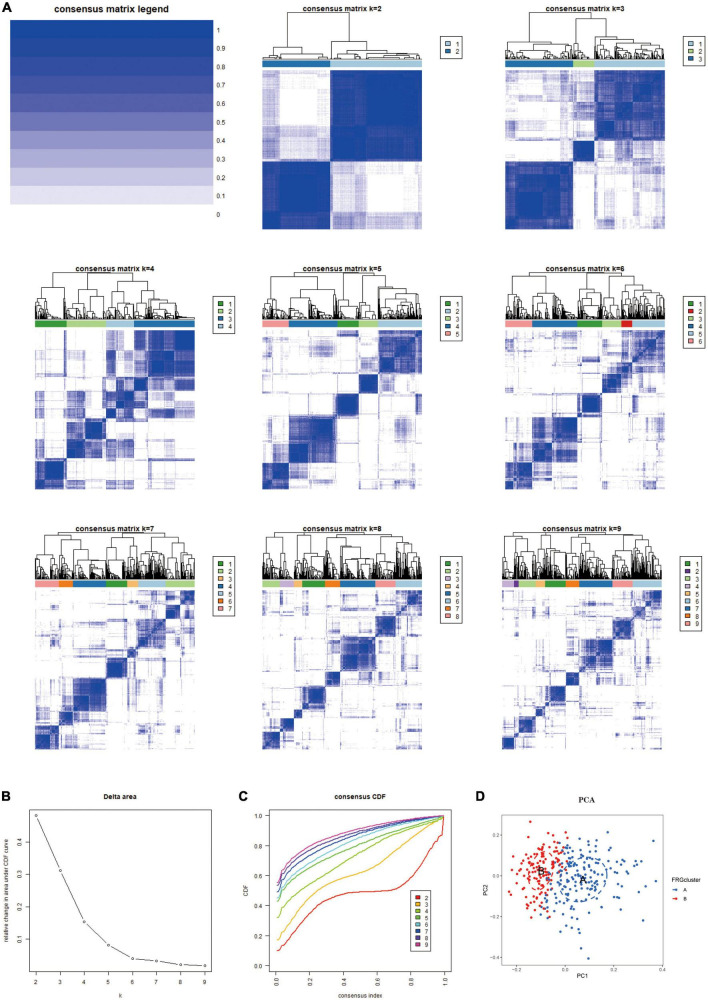
The results of 2 clusters of patients with AD. **(A)** Heatmap of consensus clustering for *k* = 2–9. Colour gradients indicate from 0 to 1 (white: 0, blue: 1). **(B)** Delta represents the relative change course in the area under the CDF curve when *k* = 2–9. **(C)** Cumulative distribution function (CDF); **(D)** PCA results of the expression profiles of the two FRGcluster patterns, showing the marked differences in the transcriptomes between the different FRGclusters. The red dots in the scatter plot represent FRGcluster A, and the blue dots represent FRGcluster B. CDF, cumulative distribution function; FRGcluster, Ferroptosis-related gene cluster.

Based on the 10 Hub genes, we performed a Consensus Cluster analysis. Specifically, the number of clusters is determined based on the results of the Consensus Matrix heatmap, the empirical cumulative distribution function (CDF) map, and the delta map. The results showed that the fractal was most stable at *k* = 2 (Figure 4A). Instead, there was a significant difference in the relative change in the area under the CDF curve when *k* = 2–9 (Figure 4B). In addition, the CDF graph (Figure 4C) curve shows a minor fluctuation when the consistency index is between 0.2 and 0.6. Based on the Consensus Cluster approach, we finally identified two FRG Clusters among 310 AD samples, of which 178 AD samples were identified as FRG Cluster A, and 132 AD samples were identified as FRG Cluster B. Principal component analysis showed (Figure 4D). There was a clear distinction between FRG Cluster A and B. We also calculated the scores of Hub genes (FRGscore) in each AD sample (Supplementary Data Sheet 3).

#### Results of immune cell infiltration analysis based on two FRG clusters

**FIGURE 5:**
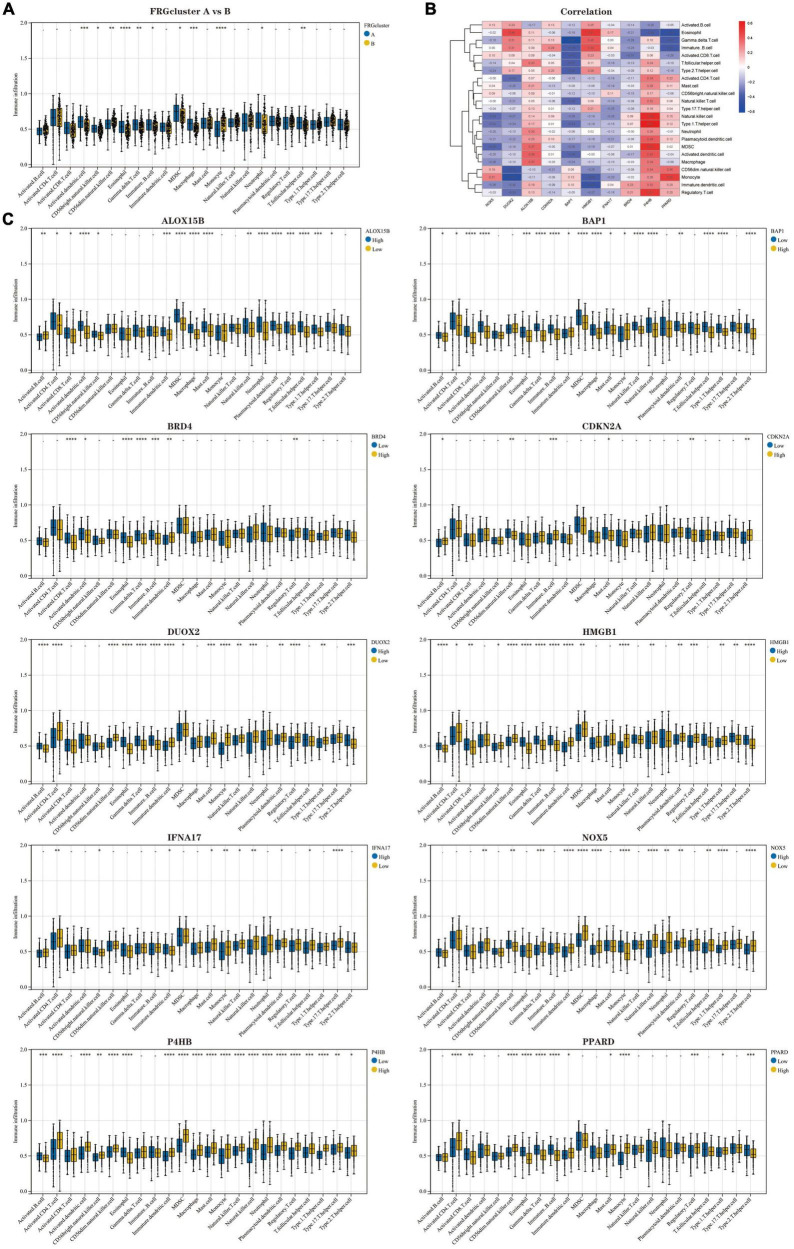
Analysis of immune cell infiltration in the two clusters of patients with AD. **(A)** Comparison of the percentage of immune cell infiltration between FRGcluster A and B. Blue represents FRGcluster A; yellow represents FRGcluster B. **(B)** Heatmap of the correlation between Hub genes and 28 immune cells with a colour gradient change from red (positive correlation) to blue (negative correlation). **(C)** Comparison of the differences in immune cell abundance between the high and low expression groups for each Hub gene. *P < 0.05; ^**^P < 0.01; ^****^P < 0.001. FRGcluster, Ferroptosis-related gene cluster.

The results of ssGSEA analysis showed (Figure 5A) (Supplementary Data Sheet 4): Activated. Dendritic.cell, CD56bright.natural.killer.cell, Eosinophil, Gamma.delta.T.cell, Immature.B.cell, MDSC, Macrophage, Neutrophil, and T.follicular.helper.cell infiltrated significantly higher proportions in FRG Cluster type A than type B, but the opposite was true for CD56dim.natural.killer.cell and Monocyte. We further analysed the correlation between 10 Hub genes and immune cells, presented in the form of a heatmap (Figure 5B). In addition, we analysed the difference in the degree of immune cell infiltration when each gene was highly and lowly expressed (Figure 5C). The results confirm an inextricable relationship between the high and low expression levels of genes and the infiltration of immune cells.

#### Results of differential expression genes between two distinct clusters

**FIGURE 6:**
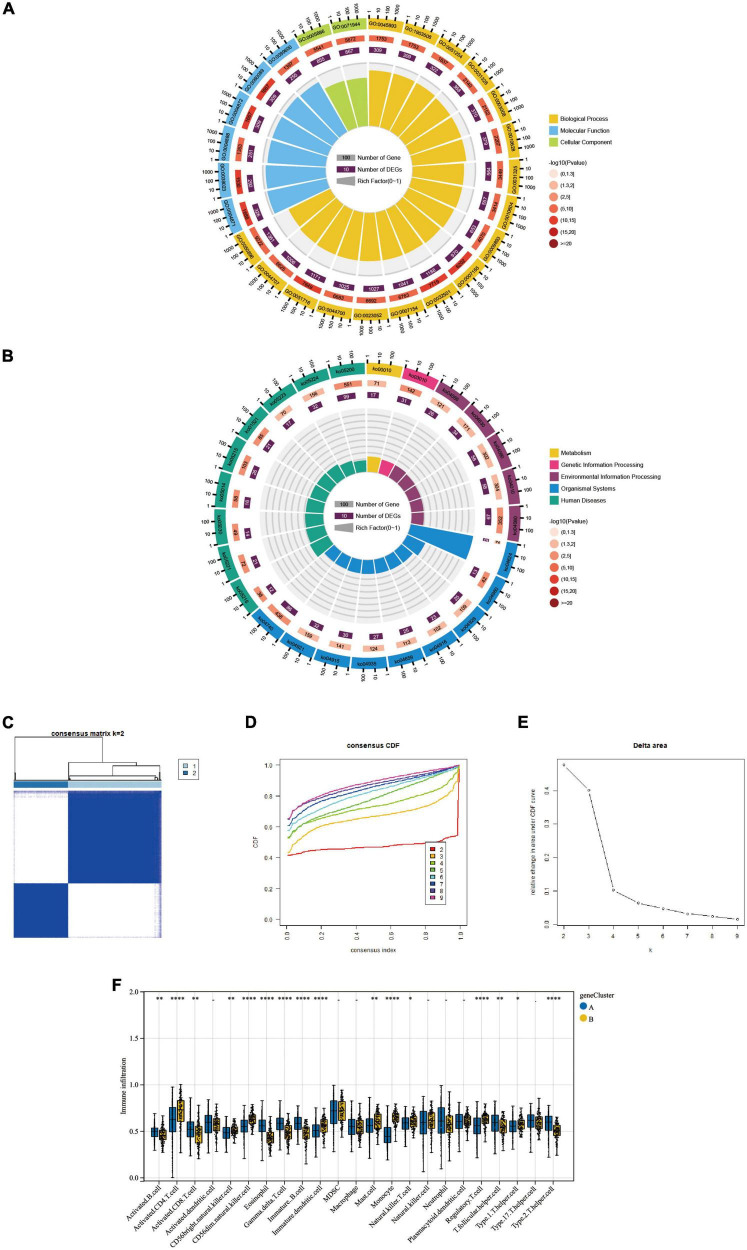
**(A,B)** The GO enrichment and KEGG pathway analysis results of CDEGs are shown as circle plots. For enrichment analysis of the circle plot, the GO id (or pathway id) label of the first circle corresponds to the “id” of the result data (Supplementary Table 1), and The “class” of the result data corresponds to the colour of the grouping. The length of the bar in the second circle corresponds to the “bg_num” of the resulting data, i.e., the number of background genes, and the shade of the colour corresponds to the *P*-value (or *Q* value). The third circle corresponds to the “fg_num” of the resulting data, i.e., the number of foreground genes. The fourth circle (polar bar) shows the Rich factor, obtained by dividing fg_num and bg_num and corresponds to the data in the ratio column of “Supplementary Table 1”. **(C)** Cluster-heatmap. **(D)** Cumulative distribution function (CDF). **(E)** Delta area plot. **(F)** Comparison of the percentage of immune cell infiltration between geneCluster A and B; Blue represents FRGcluster A; yellow represents FRGcluster B. *P < 0.05; ^**^P < 0.01; ^****^P < 0.001. CDEGs, cluster differential expressed genes; GO, Gene Ontology; KEGG, Kyoto Encyclopedia of Genes and Genomes; CDF, cumulative distribution function.

By comparing the two molecular typings of FRG Cluster A and B, we identified 3313 differentially expressed genes (CDEGs). And the GO and KEGG enrichment analysis was performed on the above-obtained CDEGs. The results showed (Table 1) that the GO enrichment analysis (Figure 6A) mainly included the positive regulation of the metabolic process, macromolecule metabolic process, cellular metabolic function and other biological pathways. The KEGG pathway shows (Figure 6B) that CDEGs exert their bodily functions mainly through the Olfactory transduction pathway. In addition, based on Consensus Cluster analysis, we established a new molecular typing based on CDEGs, defined as a “gene Cluster.” AD patients were classified into two subtypes, gene Cluster A and B, by cluster heatmap (Figure 6C), CDF plot (Figure 6D) and Delta plot (Figure 6E) selection. In addition, we also used the ssGSEA algorithm, and we derived the difference in the degree of infiltration of 28 immune cells in the two gene Clusters (Figure 6F).

#### Association of FRGscore with clinical and immunological characteristics of different subtypes

**FIGURE 7:**
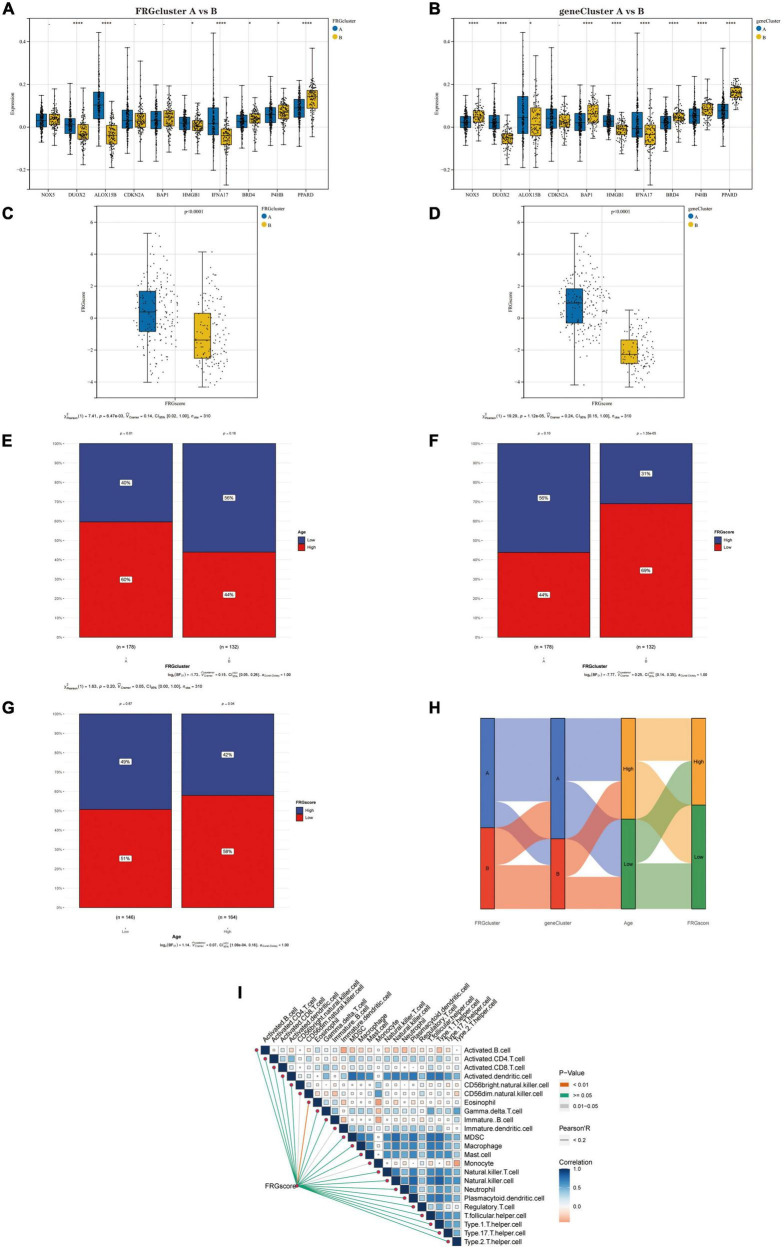
**(A)** Differential expression of Hub genes between FRGcluster A and B. **(B)** Differential expression of Hub genes between geneCluster A and B. **(C)** The corresponding FRGscore was obtained based on the PCA algorithm to compare the similarities and differences between the FRGcluster A and B subtypes FRGscore values. **(D)** The corresponding FRGscore was obtained using the PCA algorithm to compare the similarities and differences between the geneCluster A and B subtypes FRGscore values. **(E)** Percentage of senior and junior populations in both FRGcluster A and B subtypes. **(F)** Percentage of high and low age groups in both FRGcluster A and B subtypes. **(G)** Age distribution of the population in the high FRGscore and low FRGscore groups. **(H)** Sanky plots indicate clinical and molecular correlations. **(I)** Correlations between FRGscore and 28 immune cells. In this figure, the red line represents *P* < 0.01, the grey line represents *P* = 0.01–0.05, and the green line represents *P* ≥ 0.05; the colour of the squares represents the correlation coefficient, with blue being a negative correlation and red a positive correlation. *P < 0.05; ^**^P < 0.01; ^****^P < 0.001. FRGcluster, Ferroptosis-related gene cluster; FRGscore, Ferroptosis-related gene score; PCA, Principal component analysis.

First, we compared the expression levels of Hub genes between the two “FRG Clusters” and the two “gene Clusters.” Four genes (DUOX2, ALOX15B, IFNA17 and PPARD) were significantly differentially expressed between type A and type B in the FRG Cluster (Figure 7A). Also, the results showed that eight genes (NOX5, DUOX2, BAP1, HMGB1, IFNA17, BRD4, P4HB and PPARD) significantly differentially expressed between type A and type B in the gene Cluster (Figure 7B). We further analysed the differences in scores between different subtypes based on the FRGscore results derived from PCA. It was found that the FRGscore of type A was higher than that of type B in the FRG Cluster (Figure 7C), and the same result was obtained in the geneCluster (Figure 7D). We observed that the FRG Cluster B group was associated with lower age (with 80 years as the cut-off) (Figure 7E) and lower FRGscore values (Figure 7F). Also, the chi-square test confirmed that a higher percentage of patients in the low FRG score group were of lower age (Figure 7G). In addition, we analysed the relationship between FRG Cluster, gene Cluster, age and FRGscore and showed it as a Sanky plot (Figure 7H). Further correlation analysis between FRG score and immune cells showed that FRG score had a significant negative correlation with Eosinophil, CD56dim.natural.killer.cells, Immature.dendritic.cells and Monocyte (Figure 7I), which implies that dysregulation of the immune microenvironment plays a crucial role in AD development.

### Part two

#### Correlation analysis between Hub genes and immune characteristics

**FIGURE 8:**
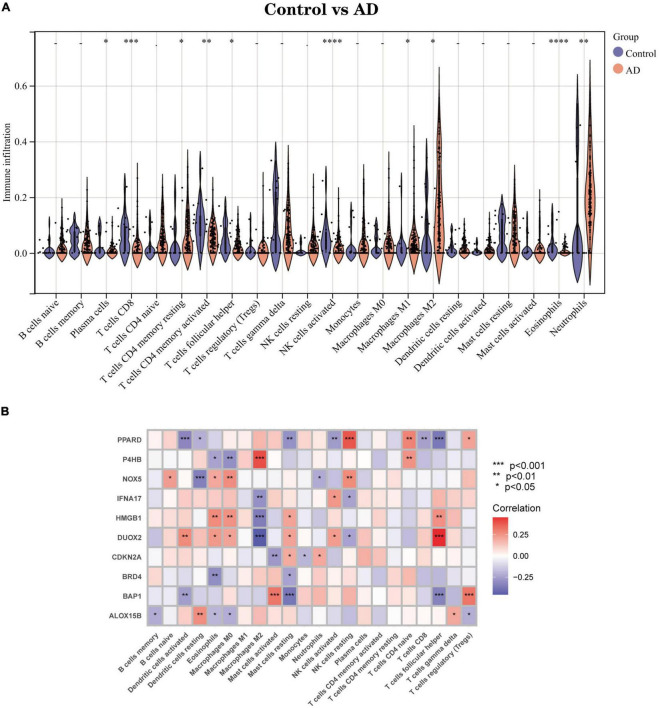
Immune infiltration landscape between AD and Control obtained by CIBERSORT analysis. **(A)** Violin plot showing the difference in immune cell infiltration between AD (red) and Control (purple), *P* < 0.05, was considered statistically significant. **(B)** Shows the correlation between Hub genes and immune cells. The colors from red to purple represent the change from positive to negative correlations, respectively. More asterisks and darker colors of the modules represent stronger correlations. *P < 0.05; ^**^P < 0.01; ^****^P < 0.001.

We used an alternative immune infiltration algorithm to explore the differences in immune infiltration between AD patients and normal samples (Supplementary Data Sheet 5). As shown in Figure 8A, the proportion of Plasma cells, T cells CD8, T cells CD4 memory resting, T cells CD4 memory activated, T cells follicular helper, NK cells activated, and NK cells activated was decreased in AD samples compared to normal samples. The percentage of infiltrated Eosinophils decreased compared with normal samples, while the opposite was true for Macrophages M1, Macrophages M2, and Neutrophils. Further analysis of the correlation of Hub genes with 22 immune cell infiltrations showed (Figure 8B) a significant positive correlation of Macrophages M2 with gene P4HB and a negative correlation with IFNA17, HMGB1 and DUOX2. Mast cells activated had a significant positive correlation with BAP1 and a negative correlation with CDKN2A; T cells follicular helper had a strong negative correlation with genes PPARD and BAP1 and a significant positive correlation with DUOX2. This evidence suggests that changes in the immune microenvironment of AD patients may be related to these 10 Hub genes.

#### Hub genes were closely linked to a variety of AD-related pathways

**FIGURE 9:**
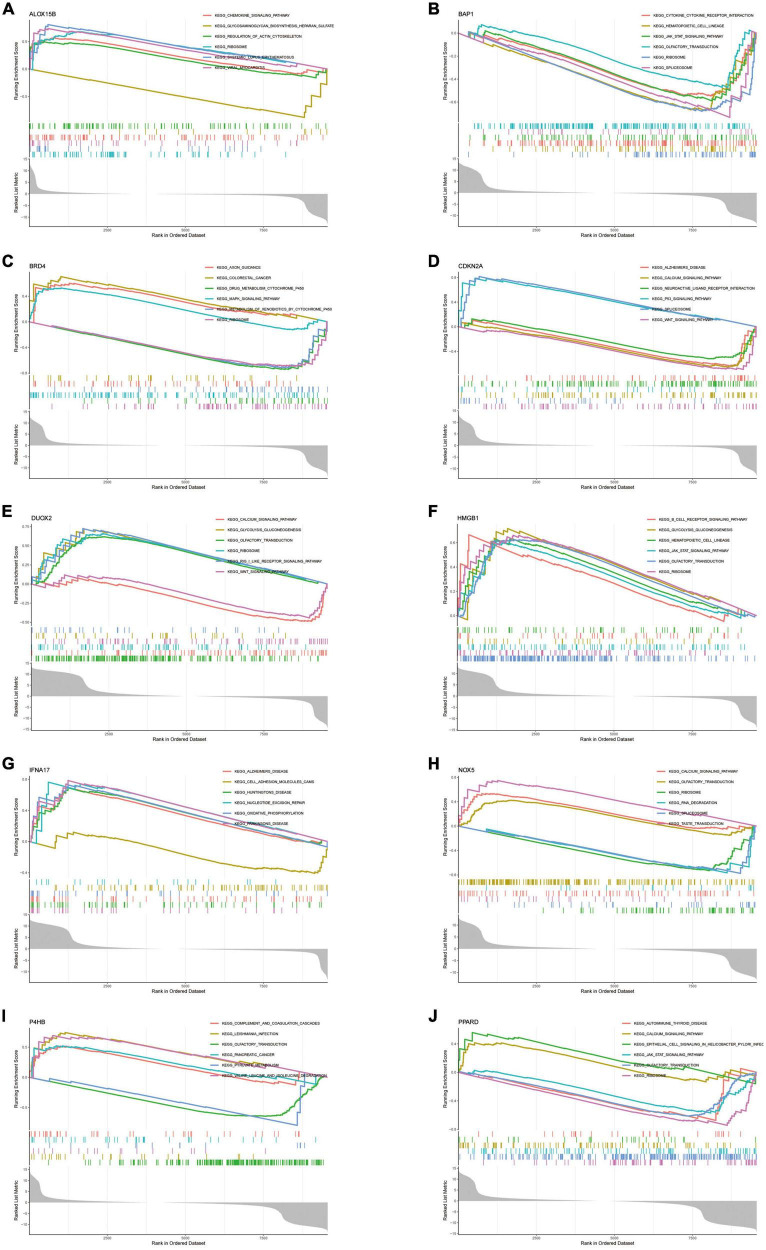
**(A–J)** Single-gene GSEA-KEGG pathway analysis in ALOX15B **(A)**, BAP1 **(B)**, BRD4 **(C)**, CDKN2A **(D)**, DUOX2 **(E)**, HMGB1 **(F)**, IFNA17 **(G)**, NOX5 **(H)**, P4HB **(I)**, PPARD **(J)**.

To explore the function of Hub genes in AD development, we performed a single-gene GSEA pathway analysis (including GO & KEGG). The results (Figures 9A–J) demonstrate the top 6 pathways for each gene enrichment. Where almost all genes are involved in the Ribosome pathway. HMGB1, BAP1, DUOX2, NOX5, P4HB and PPARD are co-enriched in the Olfactory transduction pathway. It is important to note that CDKN2A and IFNA17 were enriched in Alzheimer’s disease. Also, the results of the enrichment analysis showed that Hub genes are also involved in the JAK-STAT pathway, Spliceosome and Wnt signalling pathway pathways. In addition, the functions of some of the genes point to the B-cell Receptor Pathway and Chemokine signalling pathway, further confirming the role of Hub in the immune microenvironment.

**FIGURE 10:**
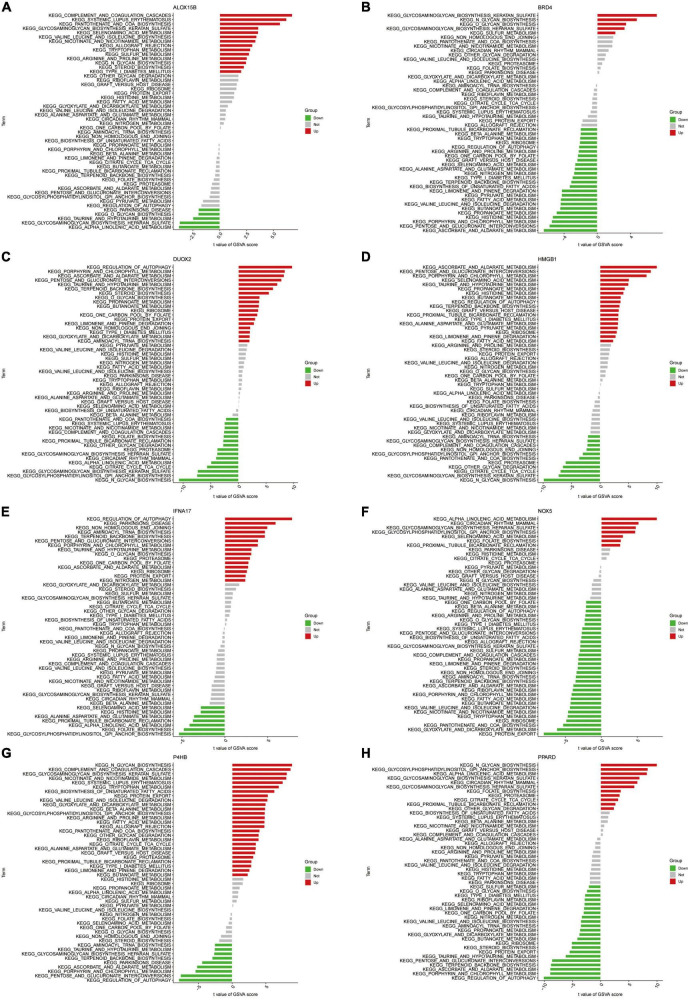
**(A–H)** High- and low-expression groups based on the expression levels of each marker gene combined with GSVA in ALOX15B **(A)**, BRD4 **(B)**, DUOX2 **(C)**, HMGB1 **(D)**, IFNA17 **(E)**, NOX5 **(F)**, P4HB **(G)**, PPARD **(H)**.

We further performed GSVA enrichment analysis of Hub genes (Figures 10A–H). Differences in the pathways activated between the gene’s high and low expression groups were predicted and observed. Results showed that the upregulation of genes NOX5 and PPARD and the downregulation of ALOX15B and IFNA17 could jointly activate the Alpha-Linolenic acid metabolism pathway. Activation of the Glycosaminoglycan biosynthesis heparan sulfate pathway is associated with the down-regulation of gene NOX5 and BRD4 expression and up-regulation of ALOX15B and P4HB expression. Increased expression levels of DUOX2 and HMGB1 and decreased expression levels of ALOX15B and P4HB affect the activation of the Taurine and hypotaurine metabolism pathways. On the other hand, the activation of Glycosylphosphatidylinositol (GPI) anchor biosynthesis depended on the upregulation of NOX5 and PPARD expression and the downregulation of IFNA17 expression levels. In addition, activation of Ascorbate and Aldarate Metabolism, Porphyrin and chlorophyll metabolism pathways were all associated with high expression of genes DUOX2 and HMGB1 with low expression of P4HB.

#### Prediction of Hub gene targeted drugs and establishment of regulatory networks

**FIGURE 11:**
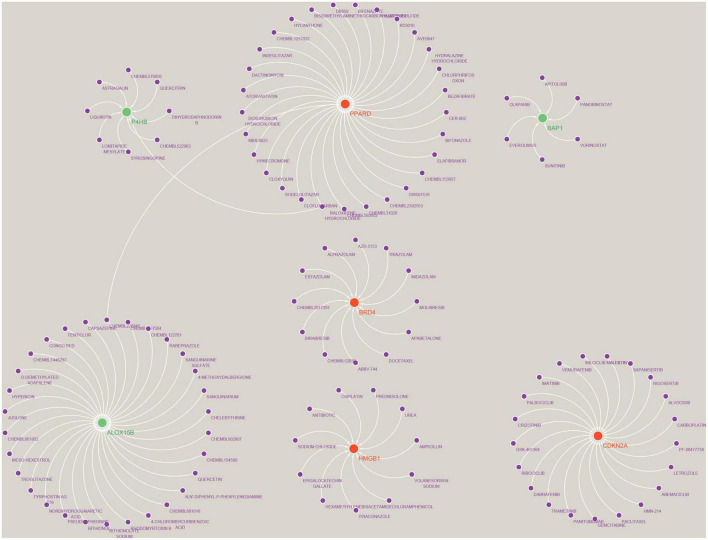
Prediction of marker gene-targeted drugs. A total of 117 drugs were retrieved that acted on the Hub gene. Of these, 30 drugs targeted ALOX15B and PPARD; 21 drugs targeted CDKN2A; 12 drugs targeted BRD4; 11 drugs acted on HMGB1; 9 drugs acted on P4HB; and 6 drugs targeted BAP1, respectively. Where red orbs represent up-regulated mRNA, green orbs represent down-regulated mRNA, and purple orbs represent drugs.

We used the Drug-Gene Interaction Database (see text footnote 2) to predict drugs that may act on the Hub gene (Supplementary Data Sheet 6) and analysed the interactions between genes and drugs (Supplementary Data Sheet 7). Finally, the results were visualised by Cytoscape, as shown in Figure 11. We retrieved a total of 117 drugs acting on the Hub gene. Of these, 30 drugs targeted ALOX15B and PPARD; 21 drugs targeted CDKN2A; 12 drugs targeted BRD4; 11 drugs acted on HMGB1; 9 drugs acted on P4HB; and 6 drugs targeted BAP1, respectively. Unfortunately, we did not predict drug targets for NOX5, DUOX2 and IFNA17.

#### Construction of ceRNA networks for Hub genes

**FIGURE 12:**
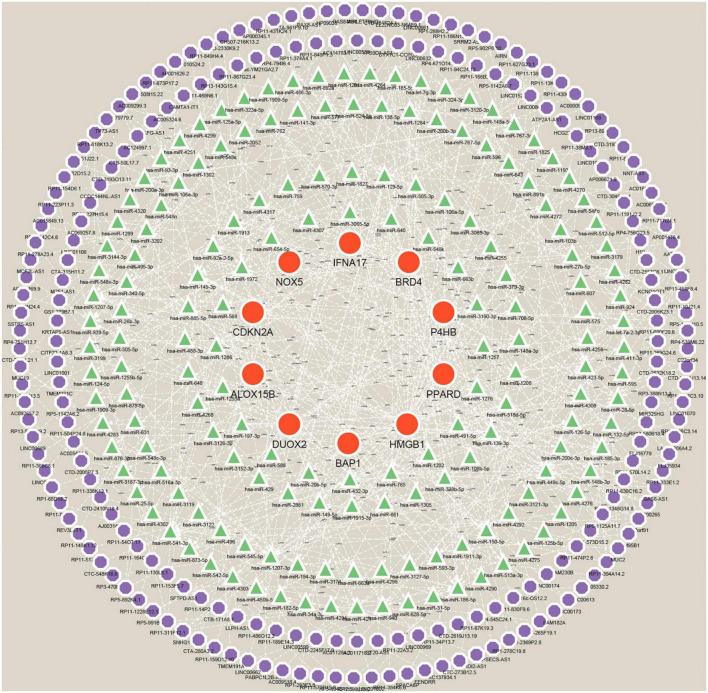
A ceRNA networks based on Hub genes. The network includes 388 nodes (10 mRNAs, 169 miRNAs, 209 lncRNAs), 503 edges. Red orbs represent Hub genes, green triangles represent miRNAs, and purple orbs represent lncRNAs.

Many studies have confirmed that ceRNA regulatory networks play a role in the biology and pathophysiology of various diseases. To explore whether Hub genes have similar regulatory relationships in AD, we established a Hub gene-based ceRNA network. The network included 388 nodes (10 mRNAs, 169 miRNAs, 209 lncRNAs) with 503 edges (Figure 12) (Supplementary Data Sheet 8). Notably, the analysis of hsa-miR-149-3p, hsa-miR-1972, hsa-miR-186-5p, hsa-miR-129-5p, hsa-miR-1207-5p, hsa-miR-541-3p, hsa-miR-185-5p, hsa-miR-939-5p and hsa-miR-449c-5p based on Degree values (Supplementary Data Sheet 9) may play a key role in the ceRNA network, regulating the expression of Hub genes. lncRNAs such as (C10orf91, HP09025, SNHG14, RP4-539M6.22, LINC00265, and MUC2) are thought to play a crucial node role in the ceRNA network.

#### Development and validation of a diagnostic model for Ferroptosis-related genes

**FIGURE 13:**
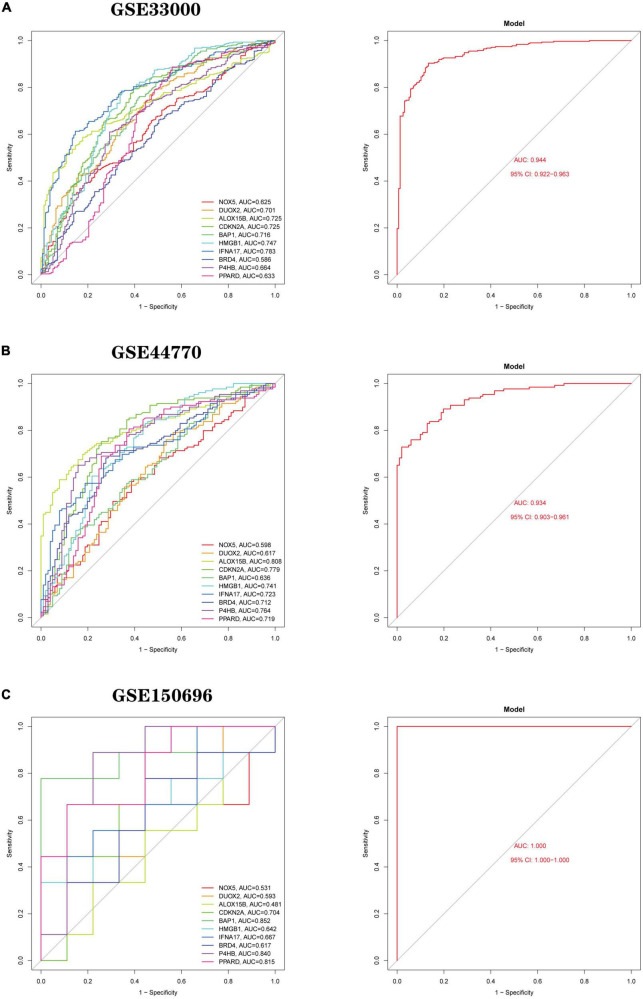
Logistic regression model building and ROC curve validation. **(A)** ROC curves of the training set GSE33000 dataset, (left) ROC curves of Hub genes, and (right) ROC curves of the model. **(B)** The validation set GSE44770 dataset, (left) ROC curve of Hub gene, (right) ROC curve of the model. **(C)** The validation set GSE150696, (left) ROC curves of Hub gene, (right) ROC curves of the model. The different coloured lines represent different genes.

**FIGURE 14:**
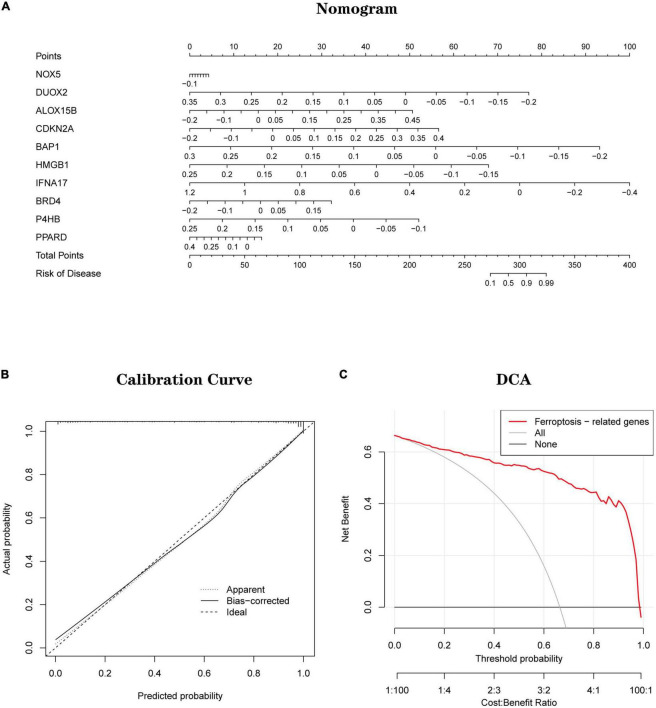
**(A)** Establishment of a nomogram for predicting the risk of AD based on feature genes in the testing set. **(B)** The calibration curve evaluates the prediction efficacy of the nomogram. **(C)** DCA estimates the clinical benefit of the nomogram.

Based on 10 Hub genes, a logistic regression algorithm was used to build a diagnostic model for AD classification. The results showed that the categorical diagnostic model built from 10 Hub genes could distinguish AD patients from normal samples. We calculated AUC values for Hub genes and models, respectively (Figure 13A): NOX5 (AUC = 0.625), DUOX2 (AUC = 0.701), ALOX15B (AUC = 0.725), CDKN2A (AUC = 0.725), BAP1 (AUC = 0.716), HMGB1 (AUC = 0.747), IFNA17 (AUC = 0.783), BRD4 (AUC = 0.586), P4HB (AUC = 0.664), PPARD (AUC = 0.633) and Model (AUC = 0.944). Furthermore, by combining these 10 Hub genes, we established Nomogram plots as a predictive tool for AD progression. In the Nomogram plot (Figure 14A), each gene corresponds to a scoring criterion, and the risk of AD progression is finally predicted by the sum of the scoring of all genes. The calibration curve of the Nomogram plot (Figure 14B) confirmed that our Nomogram plot constructed using 10 genes had a good predictive performance. In addition, the decision curve (Figure 14C) analysis demonstrated a higher clinical benefit for patients from Nomogram through the combined scoring of the 10 Ferroptosis-related Genes. A better net clinical benefit represents a better clinical application.

Finally, we use two independent datasets (GSE44770 and GSE150696) to validate the model’s accuracy. AUC values for genes and models were calculated using the same method. In the GSE44770 dataset (Figure 13B): NOX5 (AUC = 0.598), DUOX2 (AUC = 0.617), ALOX15B (AUC = 0.808), CDKN2A (AUC = 0.779), BAP1 (AUC = 0.636), HMGB1 (AUC = 0.741), IFNA17 (AUC = 0.723), BRD4 (AUC = 0.712), P4HB (AUC = 0.764), PPARD (AUC = 0.719) and Model (AUC = 0.934). In the GSE150696 dataset (Figure 13C): NOX5 (AUC = 0.531), DUOX2 (AUC = 0.593), ALOX15B (AUC = 0.481), CDKN2A (AUC = 0.704), BAP1 (AUC = 0.852), HMGB1 (AUC = 0.642), IFNA17 (AUC = 0.667), BRD4 (AUC = 0.617), P4HB (AUC = 0.840), PPARD (AUC = 0.815) and Model (AUC = 1.000). In summary, the developed model distinguishes AD patients from normal samples.

## Discussion

Ferroptosis is an iron-dependent programmed cell death that plays a vital role in the pathogenesis of AD. Several studies (Good et al., 1992; Tao et al., 2014) have confirmed the selective accumulation of iron in Aβ aggregates and neuronal fibre tangles in the brains of AD patients. Excessive iron accumulation was significantly correlated with rapid cognitive decline in AD patients (Ayton et al., 2020). On the other hand, some convincing evidence suggests (Simmons et al., 2007; Rossi et al., 2013; Mi et al., 2019; Ren et al., 2020; Wu et al., 2020) that related molecular patterns generated during Ferroptosis (e.g., ROS) induce microglia activation through the activation of neuroimmune pathways, ultimately leading to the development of neuroinflammation. This suggests that Ferroptosis-induced neuroimmune responses are an essential part of the pathogenesis of Alzheimer’s disease. Given the above evidence, an in-depth analysis of Ferroptosis-related molecular patterns and their immunological features is needed to provide a reliable research direction for future experimental studies and a theoretical basis for complementary AD biomarkers to facilitate the diagnosis and treatment of AD.

In this study, we obtained 728 Ferroptosis-related genes through the FerrDb database. Two machine learning algorithms (LASSO and mSVM-RFE) were used to select the above genes. Machine learning algorithms are widely used in many clinical areas with good results. The LASSO algorithm and the mSVM-RFE algorithm have been applied to the AD field for early AD prediction (Liu et al., 2021; Zhang et al., 2021). Through the analysis of the joint algorithm, we finally obtained 10 genes most associated with AD as Hub genes (NOX5, DUOX2, ALOX15B, CDKN2A, BAP1, HMGB1, IFNA17, BRD4, P4HB, PPARD). DUOX2 (Samadi et al., 2011), CDKN2A (Tedde et al., 2011; Antonell et al., 2016), HMGB1 (Alabed et al., 2021; Tanaka et al., 2021; Gao et al., 2022), BRD4 (Nikkar et al., 2022; Zhang et al., 2022) and PPARD (Holzapfel et al., 2006; Helisalmi et al., 2008) have been experimentally validated in the pathogenesis of AD, but NOX5, ALOX15, B, BAP1, IFNA17 and P4HB have never been studied to confirm their association with AD. The current study demonstrates that NOX5 is highly expressed in the CNS and that upregulation of its expression level can induce inflammation by mediating COX2 activation or the PG pathway (Marqués et al., 2020). In this context, it has been demonstrated that activation of the COX2 path can lead to the accumulation of ROS after ageing (Chung et al., 2009) and stroke (Duan et al., 2021). A recent study has shown that endothelial NOX5 expression alters the integrity of the blood-brain barrier and causes memory loss in ageing mice (Cortés et al., 2021). These results suggest that NOX5 may lead to AD development by mediating the immune response and thus disrupting the blood-brain barrier. Little research has been done on ALOX15B, and the current studies are limited to suggesting that ALOX15B has a role in promoting the progression of atherosclerosis (Gertow et al., 2011; Magnusson et al., 2012). Some studies have also shown its involvement in the pathogenesis of multiple sclerosis by mediating immune defence and inflammatory responses (Jäckle et al., 2020). This all suggests the potential value of ALOX15B in neuroimmune. P4HB and BAP1 are proteins involved in protein ubiquitination/deubiquitination. In the current study, BAP1 was associated with selective regional vulnerability in Parkinson’s brain (Keo et al., 2020). Studies also confirm that BAP1 may be a potential drug target for neurodegeneration (Sharma et al., 2020). P4HB (Jin et al., 2002), on the other hand, was shown to be expressed at significantly reduced levels under ischemic and hypoxic conditions in neuronal cells. We speculate that P4HB may have a protective effect on hypoxic neuronal cells through the ubiquitination pathway. IFNA17 is a protein-coding gene, and polymorphisms in the IFNA17 gene are currently thought to play a central role in the pathogenesis of multiple sclerosis (MS) (Miterski et al., 1999; Nyström et al., 2007). Still, its association with AD is poorly understood. More studies are needed to confirm it. We further analysed the function of Hub genes by integrating the algorithm analysis. We observed that almost all genes are involved in the Ribosome pathway, and ribosomal dysfunction (Ding et al., 2005; Nyhus et al., 2019) is one of the early manifestations of AD. Notably, HMGB1, BAP1, DUOX2, NOX5, P4HB and PPARD were co-enriched in the Olfactory transduction pathway, which may be related to the mechanism of olfactory impairment in AD patients. Through GSEA enrichment analysis, we observed that CDKN2A, DUOX2, NOX5 and PPARD are involved in the regulation of Calcium signalling pathways through a complex mechanism. A previous calcium hypothesis (Popugaeva et al., 2018) for AD suggested that abnormal conduction of calcium signalling pathways in neuronal cells during the early stages of the disease is the critical mechanism triggering synaptic dysfunction and neurodegeneration. This indirectly confirms our suspicions.

In a further analysis of the Hub gene, we classified the samples of patients in the AD group into FRG Cluster A and B types by cluster analysis. By ssGSEA immune infiltration algorithm analysis, we observed that Activated. Dendritic.cell, CD56bright.natural.killer.cell, Eosinophil, Gamma.delta.T.cell, Immature.B.cel, MDSC Macrophage, Neutrophil, and T.follicular.helper.cell infiltrated significantly higher proportions in FRG Cluster type A than type B, but the opposite was true for CD56dim.natural.killer.cell and Monocyte. Such results suggest that type A may be the stage of AD deterioration due to neuroinflammation, while type B is still in the period of mild inflammatory damage, which helps us to distinguish the severity of AD patients in clinical practice. Notably, Hub gene expression levels also differed between types A and B, suggesting the potential value of Hub gene-based typing in identifying the degree of disease progression in AD patients. In addition, we further analysed the differences in scores between subtypes based on the FRGscore results derived from PCA. Higher FRGscore were found for type A than type B in the FRG Cluster, and it was observed that type B seemed to be associated with lower age and lower FRGscore. Meanwhile, further correlation analysis between FRG score and immune cells showed that FRG score had a significant negative correlation with Eosinophil, CD56dim.natural.killer.cells, Immature.dendritic.cells and Monocyte, which implies that dysregulation of the immune microenvironment plays a crucial role in AD development. This confirms the complex regulatory relationship between molecular mechanisms related to Ferroptosis and immunity and suggests that our genotyping based on Hub gene construction can effectively identify AD patients who are still in the early stages of inflammation.

Subsequently, we performed a correlation analysis between Hub genes and immune cells using the CIBERSORT algorithm. The results showed that Macrophages M2 was significantly and positively correlated with gene P4HB and negatively correlated with IFNA17, HMGB1 and DUOX2; Mast cells activated was significantly and positively correlated with BAP1 and negatively correlated with CDKN2A; T cells follicular helper was strongly and negatively correlated with gene PPARD and BAP1 and significantly and positively correlated with DUOX2. This evidence further suggests that Hub genes are involved in regulating the immune microenvironment in AD patients. In parallel, we predicted and constructed a Hub gene-based Gene-Drug regulatory network, which provides a theoretical basis for developing targeted immunotherapies for AD. Considering that miRNAs and lncRNAs may regulate mRNAs, we constructed a Hub gene-based ceRNA network on this basis to better understand the molecular regulatory mechanisms.

Finally, based on a logistic regression algorithm, we constructed a diagnostic model using Hub genes and validated the model’s accuracy in two independent datasets. Also, Nomogram plots were established as a predictive tool for AD progression. Combined with the calibration curves of the Nomogram plots, we observe a good agreement between the predicted and actual observed values. In addition, decision curve analysis demonstrates that patients can derive higher clinical benefits from Nomogram. A better net clinical benefit represents a better clinical application.

However, there are some limitations to our study. First, more clinical information needs to be considered to increase the clinical predictive value of Nomogram plots. Second, we have used multiple large sample datasets to determine and validate the accuracy of the diagnostic model. But a prospective cohort is necessary to decide on its diagnostic performance further. This will be the most critical aspect of our future research. We hope to complete it in further work to understand better the role of Ferroptosis-related molecular immune mechanisms in the pathogenesis of AD.

## Conclusion

We comprehensively analysed Ferroptosis-related molecules and their immunological features by integrating algorithmic analysis. Two different FRG clusters were successfully identified to help us differentiate the severity of AD patients in the clinical setting. Secondly, we constructed the Gene-Drug regulatory network and the ceRNA regulatory network, allowing us to better understand AD’s molecular regulatory mechanisms and reveal possible drug targets. Finally, based on the logistic regression algorithm, we built the Nomogram graph and the diagnostic model of AD. Our study provides new insights into the role of Ferroptosis and its molecular immune mechanisms in AD, as well as a theoretical basis for adding diagnostic markers for AD.

## Data availability statement

The original contributions presented in this study are included in the article/Supplementary material, further inquiries can be directed to the corresponding author.

## Author contributions

Y-JH and LC completed the design and wrote the manuscript for this study. S-LL preprocessed the data used in this study and provided suggestions for data analysis. XM, J-NT, and HL critically revised the manuscript and provided recommendations for data analysis. YW offered constructive suggestions for this study. All authors contributed to the article and approved the submitted version.

## Footnotes

1
https://www.perl.org/

2
https://dgidb.genome.wustl.edu/

## Supplementary material

Supplementary Figure 1 Hub gene expression levels in various human tissues.

Supplementary Figure 2 Hub gene expression levels in various human tissues.
