# Supplementary figures and images for: Discovery and validation of Ferroptosis-related molecular patterns and immune characteristics in Alzheimer’s disease
Source: Front Aging Neurosci. 2022 Nov 23;14:1056312. doi: 10.3389/fnagi.2022.1056312 (PMC9727409; doi:10.3389/fnagi.2022.1056312)

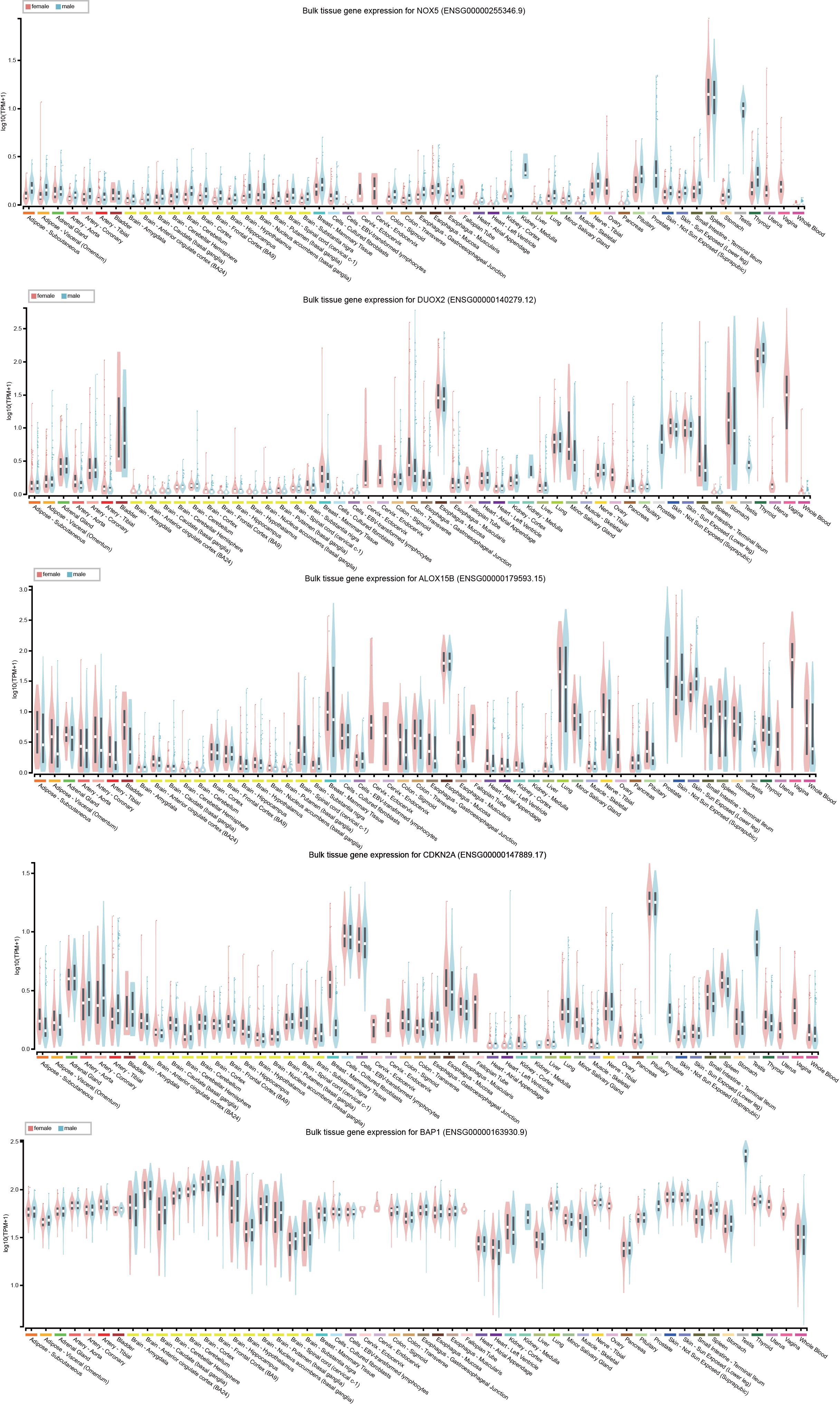

Supplement: Supplementary Figure 1 — Hub gene expression levels in various human tissues. [file Image_1.JPEG]

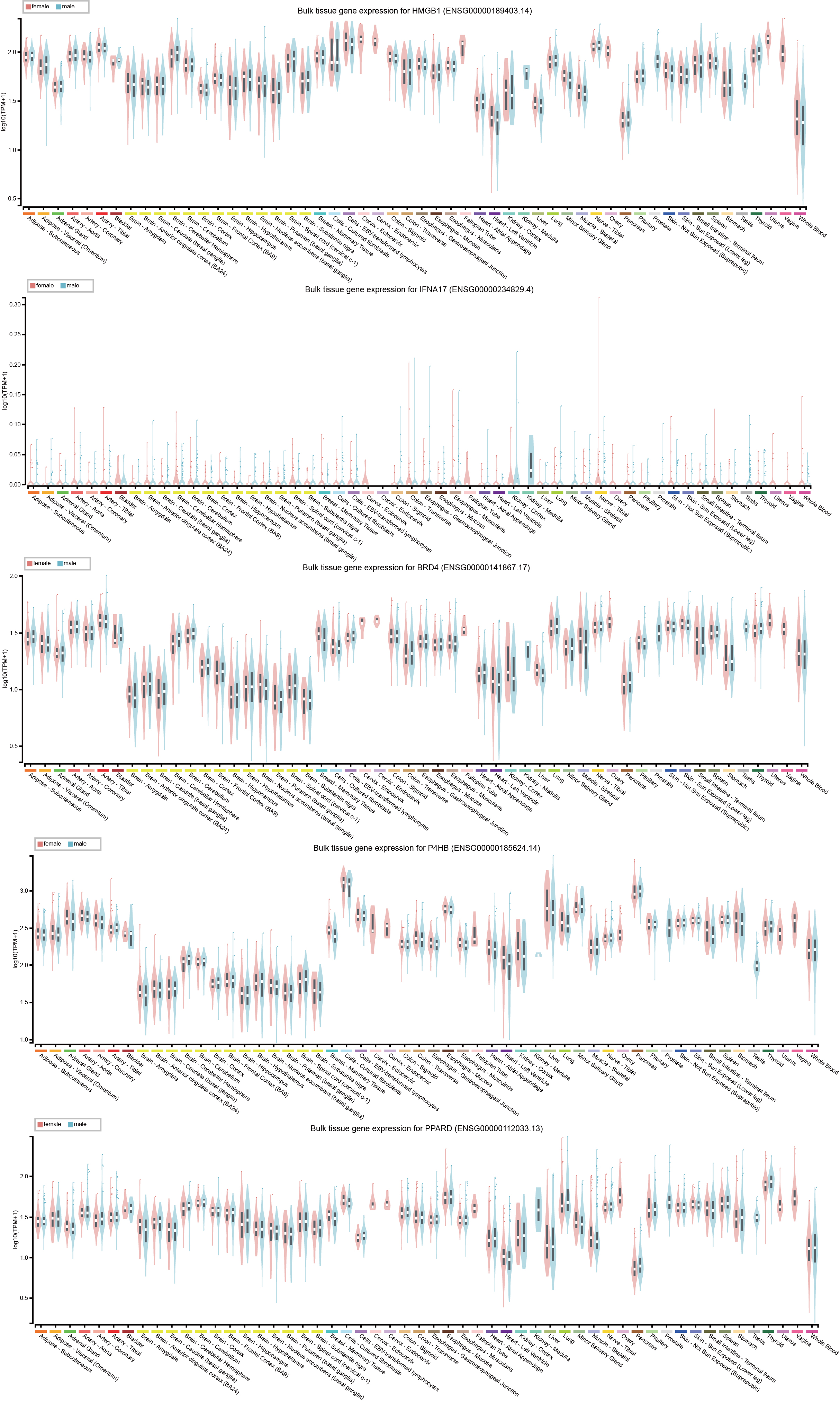

Supplement: Supplementary Figure 2 — Hub gene expression levels in various human tissues. [file Image_2.JPEG]
